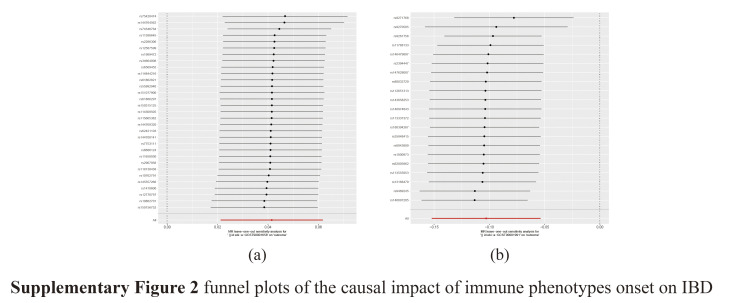# Causal role of immune cells in inflammatory bowel disease: A Mendelian randomization study

**DOI:** 10.1097/MD.0000000000037537

**Published:** 2024-04-05

**Authors:** Haoyu Chen, Qi Li, Tianyu Gao, Yuhua Wang, Xuetong Ren, Shaowei Liu, Shixiong Zhang, Pingping Zhou, Jingjing Lyu, Haiyan Bai, Yangang Wang

**Affiliations:** a School of Graduate, Hebei University of Chinese Medicine, Hebei, Shijiazhuang, China; b School of Graduate, Nanjing University of Chinese Medicine, Nanjing, Jiangsu, China; c Department of Gastroenterology, Hebei Province Hospital of Chinese Medicine, Shijiazhuang, China; d Department of Gastroenterology, Beijing University of Chinese Medicine, Third Affiliated Hospital, Beijing, China.

**Keywords:** immunity, inflammatory bowel disease, Mendelian randomization, single-nucleotide polymorphism

## Abstract

Inflammatory bowel disease (IBD) is characterized by an inflammatory response closely related to the immune system, but the relationship between inflammation and IBD remains unclear. We performed a comprehensive 2-sample Mendelian randomization (MR) analysis to determine the causal relationship between immune cell characteristics and IBD. Using publicly available genetic data, we explored the relationship between 731 immune cell characteristics and IBD risk. Inverse-variance weighting was the primary analytical method. To test the robustness of the results, we used the weighted median-based, MR-Egger, simple mode, and mode-based methods. Finally, we performed a reverse MR analysis to assess the possibility of reverse causality. We identified suggestive associations between 2 immune cell traits and IBD risk (*P* = 4.18 × 10^–5^ for human leukocyte antigen-DR on CD14+ monocytes, OR: 0.902; 95% CI: 0.859–0.947; for CD39+ CD4+ T cells, *P* = 6.24 × 10^–5^; OR: 1.042; 95% CI: 1.021–1.063). Sensitivity analysis results of these immune cell traits were consistent. In reverse MR analysis, we found no statistically significant association between IBD and these 2 cell traits. Our study demonstrates the close connection between immune cells and IBD using MR, providing guidance for future clinical and basic research.

## 1. Introduction

Inflammatory bowel disease (IBD) is a chronic, nonspecific inflammatory disease of the intestinal tract, the cause of which is not well understood and is mainly categorized as ulcerative colitis or Crohn disease.^[1–3]^ With continuous industrialization, the incidence and prevalence of IBD are rapidly increasing worldwide.^[4]^ Asia, with an aging population, is expected to experience an exponential increase in the number of patients with IBD.^[5]^ In addition, IBD is a lifelong progressive disease that imposes a significant burden on individuals, families, and society.^[6,7]^ Current research suggests that its pathogenesis may be related to a variety of factors, such as infection, environment, genetics, immunity, and gut microbes, with the immune system receiving increasing attention in the pathogenesis of IBD.^[8,9]^

The gut is the largest immune organ in the body, and both adaptive and innate immunity play important roles in the pathogenesis of IBD.^[3,10]^ Studies have shown that immune cells such as T cells, innate lymphoid cells, dendritic cells (DC), and macrophages are important links in IBD.^[11,12]^ T cells can adapt to the immune microenvironment and form complex networks of interactions with other immune cells that regulate the further progression of IBD.^[13]^ Th17 cells regulate the immune microenvironment of the intestinal mucosa by secreting cytokines, such as IL-17, IL-21, and IL-26, which in turn affect IBD progression.^[14]^ Type 2 innate lymphoid cells influence the development and progression of IBD by modulating chronic immunity and inflammation.^[15]^ DCs and regulatory T cells (Treg) influence the inflammatory response of intestinal epithelial cells by modulating immune homeostasis and tolerance in the gut.^[16]^ Macrophages have a crucial influence on the inflammatory state, with pro- and antiinflammatory functions, and M1 and M2 macrophages participate together in the pathological process of IBD, playing an important role in the M1/M2 balance.^[17,18]^ In addition, many inflammatory factors such as IL-1, IL-10, IL-12, IL-23, IL-33, IL-36, and IL-38, which are involved in regulating the homeostasis of intestinal immunity and act as pro- and antiinflammatory agents, also play a crucial role in the development of IBD.^[19–23]^ However, the relationship between immune inflammation and IBD remains unclear, which may possibly be due to limited sample sizes, the complexity of the underlying mechanisms, and confounding factors outside the scope of existing studies.^[3,24]^

Mendelian randomization (MR) refers to an analytical method used to assess the causal relationship between an exposure or risk factor and a clinically relevant outcome.^[25,26]^ MR studies have been widely conducted in medical research using genetic variations, typically single-nucleotide polymorphisms (SNPs), to eliminate confounding factors.^[27]^ Currently, many studies have demonstrated the association between immune cell traits and IBD, supporting the hypothesis that there is a correlation between them.^[3,10,28,29]^ However, whether immune cell traits are responsible for the development of IBD remains unclear. High-level evidence on a causal relationship between immune cell traits and IBD is urgently needed to guide clinical decision-making and provide an orientation for basic research. Therefore, in this study, we performed a comprehensive 2-sample MR analysis to identify the causal relationship between immune cell traits and IBD and to determine whether immune cell traits are responsible for the occurrence of IBD and the strength of their effect.

## 2. Materials and methods

### 2.1. Design of study

**Figure 1.**
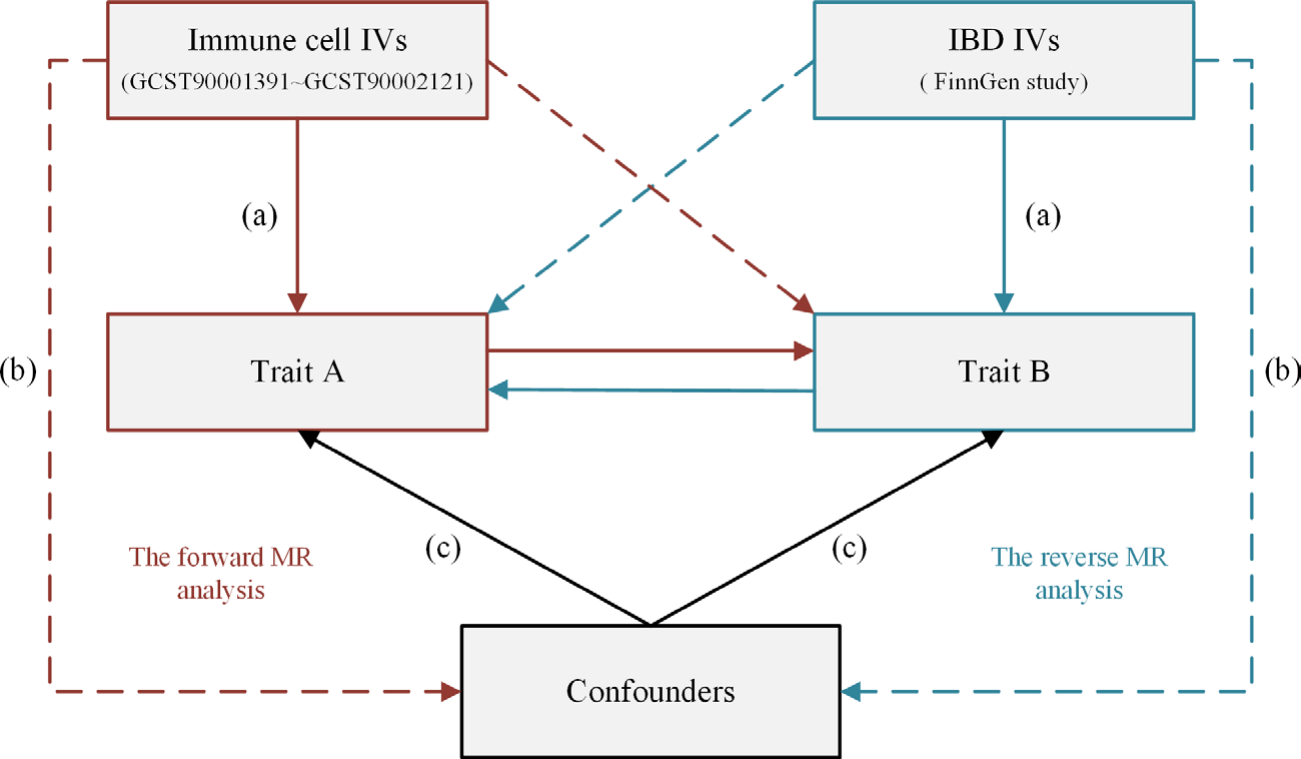
Schematic representation of the bidirectional 2-sample MR design for studying the relationship between immune cell traits and IBS. The red analyses examine the causal effect of immune cell traits as an exposure factor on IBS as an outcome, while the blue analyses examine the reverse association. To be valid tools, the genetic variants used in the study must meet 3 criteria: (A) The stable and significant correlation between genetic variation and exposure factors. (B) The confounders affecting the relationship between exposure factors and outcomes do not correlate with genetic variation. (C) Exposure factors are the only way in which genetic variation affects outcomes, not through other routes. IBD = inflammatory bowel disease, IVs = instrumental variables, MR = Mendelian randomization.

We performed bidirectional 2-sample MR studies to assess the causal relationship between immune cell traits and IBD (Fig. 1). MR uses genetic variation to represent risk factors; therefore, effective instrumental variables (IVs) in causal inference must satisfy 3 key assumptions^[30,31]^: the correlation hypothesis, which indicates a stable and significant correlation between genetic variation and exposure factors; the independence hypothesis, which states that confounders affecting the relationship between exposure factors and outcomes do not correlate with genetic variation; and the exclusion of the restrictive hypothesis, which asserts that genetic variation affects outcomes only through exposure factors and not through other routes.

### 2.2. Summary of immune cell statistics from genome-wide association study (GWAS)

Summary information for each immune cell type was obtained from the GWAS statistical data. This information is publicly available from the GWAS Catalog (accession numbers GCST90001391–GCST90002121).^[32]^ We summarized GWAS investigations of immune cell traits, including 731 immune phenotypes. Flow cytometry analyses were performed in a general population cohort, which included the absolute cell count (n = 118), median fluorescence intensity reflecting surface antigen levels (n = 389), morphological parameters (n = 32), and relative cell count (n = 192). The cell types included in the research were T, B, and natural killer cells; maturation stages of T cells; Tregs; DCs; B cells; monocytes; and myeloid cell panels. In this study, a GWAS was performed by determining the Sardinian sequences on a reference panel of 3514 individuals and testing 20,143,392 SNPs and 1688,858 indels.

### 2.3. Summary of IBD statistics from FinnGen

The IBD data for IBD came from the R9 dataset published by the FinnGen study^[33]^ with a total sample size of 369,652, a case group of 7625, and more than 20 million SNPs.

### 2.4. Selection criteria for IVs

The significance level of the IV for each immune signature was set to 1 × 10^–5^, according to recent studies.^[32,34,35]^ These SNPs (linkage disequilibrium *r*^2^ threshold <0.1 within 500 kb distance) were trimmed using the clump function in the R package TwoSampleMR,^[36]^ where linkage disequilibrium *r*^2^ was a control calculated according to the 1000 Genomes Project as a reference plate. For IBD, the data were screened and analyzed to enhance the reliability of the results (*P* < 5 × 10^–8^) by referring to multiple publications and developing stricter criteria.^[37,38]^ Meanwhile, we calculated the proportion of phenotypic variation explained and F-statistics for each IV to assess IV strength and avoid weak instrument bias. After removing IVs with low F-statistics (<10), 21 IVs of IBD were retained for further analysis.

### 2.5. Statistical analysis

**Figure 2.**
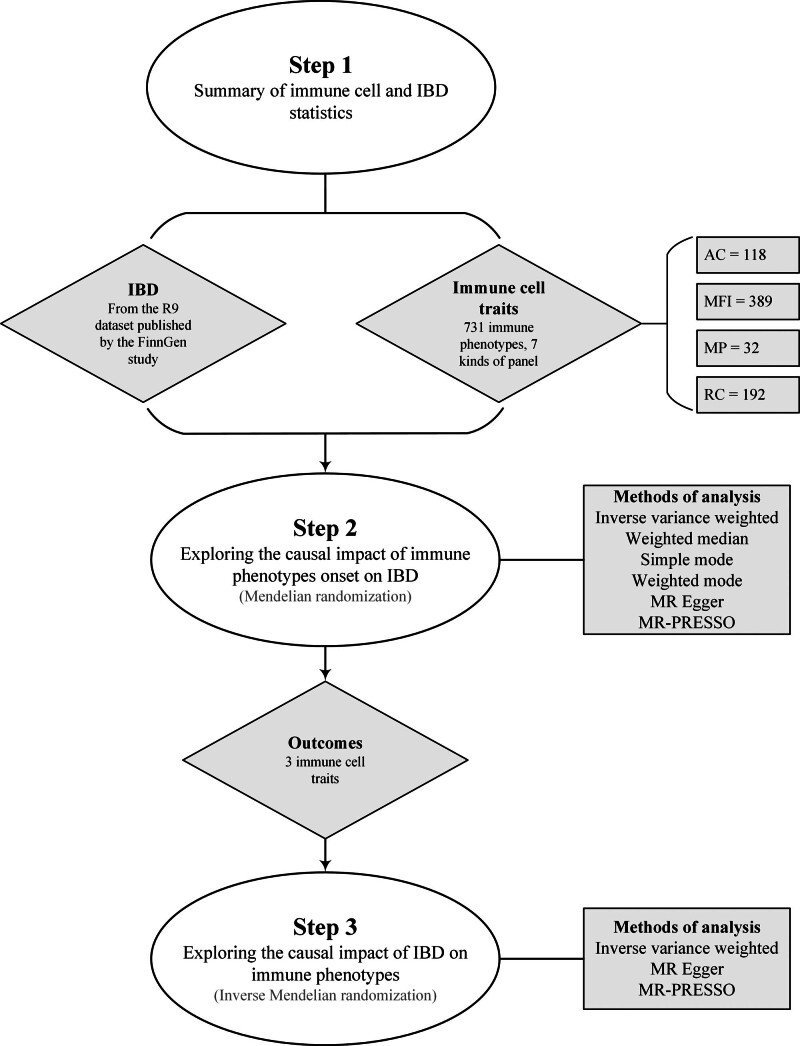
Study design for the correlation of immune cell traits with IBD. AC = absolute cell, IBD = inflammatory bowel disease, MFI = median fluorescence intensity, MP = morphological parameters, MR = Mendelian randomization, MR-PRESSO = MR pleiotropy residual sum and outliers, RC = relative cell.

Analyses were performed using R software (version v4.3.1, https://cran.r-project.org/). We evaluated the causal relationship between 731 immune phenotypes and IBD, mainly using the “TwoSampleMR” package^[36]^ to perform inverse-variance weighting (IVW),^[39]^ weighted median-based,^[40]^ MR-Egger,^[41]^ simple mode,^[42]^ and mode-based methods.^[43]^ The MR-Egger algorithm was used to eliminate the influence of horizontal pleiotropy.^[41]^ If the intercept term was significant, horizontal multiplicity existed. Furthermore, we employed the MR pleiotropy residual sum and outliers (MR-PRESSO) method to exclude possible horizontal pleiotropy outliers that could seriously affect the estimation results using the MR-PRESSO package.^[31]^ Scatter plots and funnel plots were constructed. The study design is illustrated in Figure 2.

## 3. Results

### 3.1. Exploring the causal impact of immune cell traits onset on IBD

At a nominal significance level, we detected 54 immune cells that were causally associated with IBD. These 54 immune cells were categorized into B cells (5 cells), classical dendritic cells (DCs) (9 cells), T cells at various maturation stages (5 cells), monocytes (11 cells), myeloid cells (10 cells), a combination of T, B, and natural killer cells (7 cells), and Treg panels (7 cells).

**Figure 3.**
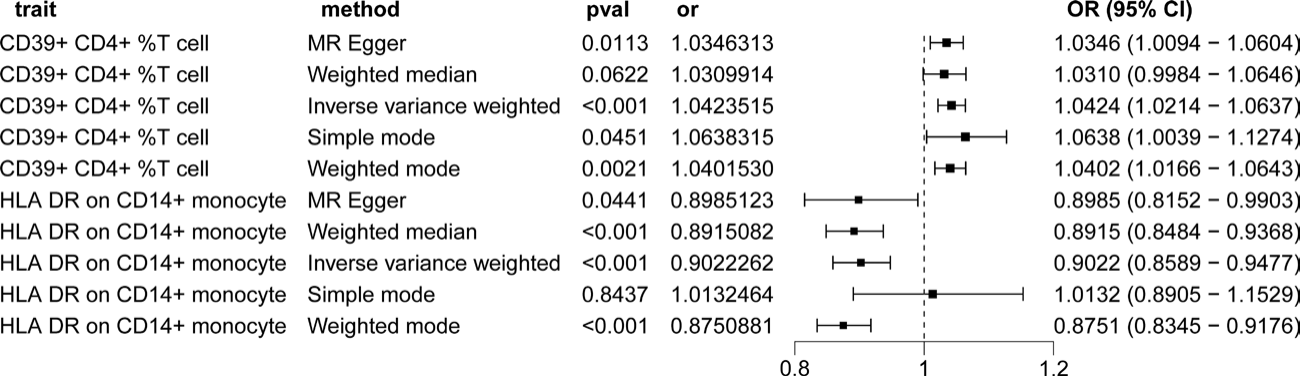
Forest plots showed the causal impact of immune cell traits onset on IBD. CI = confidence interval, OR = odds ratio.

To obtain more reliable results, we corrected and screened the *P* values using the Bonferroni correction (*P*_*Bonferroni*_ < .05/731 × 1 = 6.84 × 10^–5^). Finally, we identified 2 immune cell characteristics associated with the risk of IBD. These were human leukocyte antigen-DR (HLA-DR) on CD14+ monocytes (monocyte panel) and CD39+ CD4+ T cells (Treg panel). Using the IVW method, the odds ratio (OR) of HLA-DR on CD14+ monocytes for the risk of IBD was estimated to be 0.902 (95% confidence interval [CI]: 0.859–0.947, *P* = 4.18 × 10^–5^; Supplementary Table S1, http://links.lww.com/MD/M12, Fig. 3). Similar results were observed by using 3 methods: MR-Egger (OR: 0.899, 95% CI: 0.815–0.99, *P* = .044); weighted median (OR: 0.892, 95% CI: 0.848–0.937, *P* = 5.58 × 10^–6^); and weighted mode (OR: 0.875, 95% CI: 0.835–0.918, *P* = 2.17 × 10^–5^). Using the IVW method, the OR of CD39+ CD4+ T cells for IBD risk was estimated to be 1.042 (95% CI: 1.021–1.063; *P* = 6.24 × 10^–5^). Similar results were observed by using 3 methods: MR-Egger (OR: 1.035, 95% CI: 1.009–1.06, *P* = .011); simple mode (OR: 1.064, 95% CI: 1.004–1.127, *P* = .045); and weighted mode (OR: 1.04, 95% CI: 1.017–1.064, *P* = .002). In addition, the possibility of horizontal pleiotropy in these 3 associations was excluded using MR-Egger intercept and MR-PRESSO global tests. The robustness of the observed causal association was also demonstrated by sensitivity analysis (Supplementary Table S2, http://links.lww.com/MD/M13). The scatter plot showed that the results were not affected by outliers, whereas the funnel plot demonstrated the robustness of the correlations and the absence of heterogeneity (Supplementary Figs. S1, http://links.lww.com/MD/M10 and S2, http://links.lww.com/MD/M11).

### 3.2. Exploring the causal impact of IBD on immune cell traits

**Figure 4.**
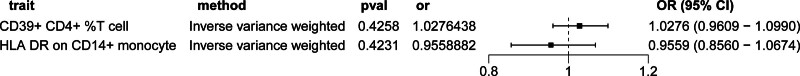
Forest plots showed the causal impact of IBD on immune cell traits. CI = confidence interval, OR = odds ratio.

Finally, the potential inverse association between these 5 immune cell traits and IBD was evaluated using reverse MR imaging. Using the IVW approach, we found no statistically significant association between IBD and any of the 5 immune cell traits (HLA-DR on CD14+ monocytes: OR: 0.956, 95% CI: 0.856–1.067, CD39+ CD4+ T cells: OR: 1.028, 95% CI: 0.961–1.099; Fig. 4). The results of the sensitivity analysis were stable, as shown in Supplementary Table S3, http://links.lww.com/MD/M14.

## 4. Discussion

Using clinical genetic data from public databases, this study analyzed the causal relationship between 731 immune cell characteristics and IBD. Using database species SNPs as IVs, we integrated a large number of 2-sample MR methods to show that 2 immune cell types, HLA-DR on CD14+ monocytes and CD39+ CD4+ T cells, were associated with IBD risk.

Our study found that HLA-DR on CD14+ monocytes was multiplicatively and negatively associated with the risk of IBD. Monocytes are closely associated with intestinal diseases.^[44]^ Studies have identified an important link between HLA-DR and IBD. HLA-DR expression plays an important role in the immunity of intestinal epithelial cells in both ulcerative colitis and Crohn disease.^[45,46]^ Intestinal epithelial cells of patients with IBD show increased proliferation rates and expression of HLA-DR, which is highly correlated with the severity of inflammation.^[47]^ CD14 is an important surface antigen of the immune system and is continuously expressed on the surfaces of monocytes, macrophages, DCs, neutrophils, and epithelial cells.^[48,49]^ CD14 affects the integrity of the intestinal barrier under inflammatory conditions, thereby playing an important role in the occurrence and development of IBD.^[50]^ In addition, a clinical study showed that CD14 is highly correlated with the level of mucosal healing in IBD and can be used as a surrogate marker for mucosal healing in IBD.^[51]^

Our study also showed that CD39+ CD4+ T cells were negatively correlated with the occurrence of IBD. As an immune switch, CD39 is expressed in various immune cells and plays an important role in a series of responses caused by the body’s immune and defense systems.^[52]^ One study showed that the number of CD39+ T cells was significantly reduced in patients with IBD, which may be related to the suppressed expression of immune function.^[53]^ In addition, animal experiments have shown that CD39 deficiency can exacerbate colitis in mice.^[54]^ As an important component of the human immune system, CD4+ T cells regulate the immune system by signaling other types of immune cells to form complex immune cell regulatory networks.^[13]^ In addition, CD4+ T cells can interact with the intestinal microbiota to affect mucosal barrier function, ultimately regulating inflammation.^[55]^ For example, fungal dysbiosis promotes IBD by enhancing CD4+ T cell glutaminolysis.^[56]^

Many previous studies have shown a connection between IBD and the immune system; however, these were observational studies.^[11,12]^ This study analyzed the causal effect of immune cell traits on IBD using genetic epidemiological methods. We used IVs whose F-statistics met a threshold of >10 and performed reverse MR analyses to rule out reverse causality. The causal association results obtained in this study provide a basis and ideas for further research on the correlation between immune cells and the occurrence and development of IBD.

This study had some limitations. First, the immune cell trait data mainly included 7 immune cell traits and did not include all immune cells. Second, the data on immune cell traits in this study were mainly from Sardinians; although there may be genetic variability, the results are still valid for generalization to other racial groups. However, to avoid possible bias, future expansion of the sample size and study population is needed for further validation. Third, we selected a loose threshold, such as *P* < 1.0 × 10^–5^, for IVs of immune cell traits that were larger than the traditional genome-wide significance level (*P* < 5 × 10^–8^) to obtain sufficient IVs. Fourth, potential residual confounders could not be completely eliminated, even after applying rigorous exclusion criteria, and requires more in-depth studies to validate the complexity of the associations. Furthermore, the effects of the immune cell traits reported were relatively weak, and no other independent IBD study had a sufficient sample size to validate our findings. Thus, further research is required to confirm this.

## 5. Conclusions

In summary, we demonstrated a causal relationship between several immune phenotypes and IBD using a comprehensive 2-way MR analysis. Furthermore, our study significantly reduced the influence of unavoidable confounders, reverse causation, and other confounding factors. The results of this study may provide new ways to explore the basic mechanisms of IBD as well as early interventions and treatments.

## Acknowledgments

All genetic data were obtained from the GWAS database and the FinnGen database. We thank all participants and researchers who provided GWAS data.

## Author contributions

**Conceptualization:** Haoyu Chen, Qi Li.

**Data curation:** Haoyu Chen, Qi Li, Tianyu Gao, Yuhua Wang, Xuetong Ren, Shaowei Liu, Shixiong ZHANG, Pingping Zhou, Jingjing Lyu.

**Formal analysis:** Haoyu Chen, Qi Li, Tianyu Gao, Pingping Zhou.

**Methodology:** Haoyu Chen, Qi Li, Tianyu Gao, Pingping Zhou.

**Project administration:** Haiyan Bai, Yangang Wang.

**Supervision:** Haiyan Bai, Yangang Wang.

**Visualization:** Haoyu Chen, Qi Li, Yuhua Wang, Xuetong Ren.

**Writing – original draft:** Haoyu Chen, Qi Li.

**Writing – review & editing:** Tianyu Gao, Shaowei Liu, Shixiong Zhang, Pingping Zhou, Jingjing Lyu, Haiyan Bai, Yangang Wang.

## Supplementary Material





**Figure:**
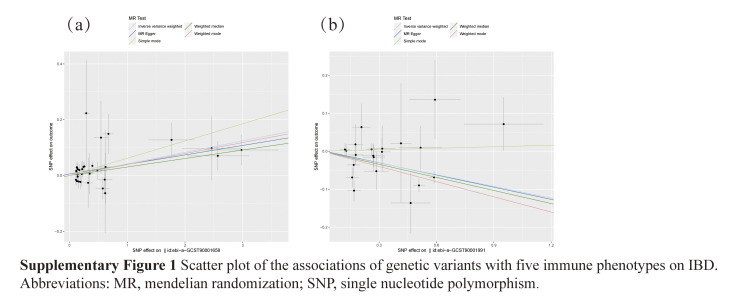


**Figure:**