# Supplementary material for: Causal role of immune cells in inflammatory bowel disease: A Mendelian randomization study
Source: Medicine (Baltimore). 2024 Apr 5;103(14):e37537. doi: 10.1097/MD.0000000000037537 (PMC10994490; doi:10.1097/MD.0000000000037537)
Supplement: Supplementary file 1 [file medi-103-e37537-s001.docx]

Supplementary Table 1 Exploring the causal impact of immune cell traits onset on IBD

| **Trait** | **id** | **method** | **pval** | **or** | **or_lci95** | **or_uci95** |
| --- | --- | --- | --- | --- | --- | --- |
| CD39+ CD4+ %T cell | ebi-a-GCST90001658 | MR Egger | 0.01125242 | 1.034631261 | 1.009441609 | 1.060449498 |
|  |  | Weighted median | 0.062191354 | 1.030991375 | 0.998445308 | 1.064598339 |
|  |  | Inverse variance weighted | 6.24E-05 | 1.042351519 | 1.02139821 | 1.063734671 |
|  |  | Simple mode | 0.045093327 | 1.063831499 | 1.003885894 | 1.12735667 |
|  |  | Weighted mode | 0.002090578 | 1.040153005 | 1.016596139 | 1.064255737 |
| HLA DR on CD14+ monocyte | ebi-a-GCST90001991 | MR Egger | 0.04412354 | 0.898512314 | 0.815212135 | 0.99032429 |
|  |  | Weighted median | 5.58E-06 | 0.891508227 | 0.848400484 | 0.9368063 |
|  |  | Inverse variance weighted | 4.18E-05 | 0.902226202 | 0.858895393 | 0.947743027 |
|  |  | Simple mode | 0.843721427 | 1.013246436 | 0.890485474 | 1.152931037 |
|  |  | Weighted mode | 2.17E-05 | 0.875088052 | 0.834503279 | 0.917646603 |
