# Supplementary material for: Causal role of immune cells in inflammatory bowel disease: A Mendelian randomization study
Source: Medicine (Baltimore). 2024 Apr 5;103(14):e37537. doi: 10.1097/MD.0000000000037537 (PMC10994490; doi:10.1097/MD.0000000000037537)
Supplement: Supplementary file 2 [file medi-103-e37537-s002.docx]

Supplementary Table 2 Sensitivity analysis results of causal effects of immune cell traits on IBD

| **Exposures** | **Outcomes** | **method** | **Q** | **Q_df** | **Q_pval** | **egger_intercept** | **se** | **pval** |
| --- | --- | --- | --- | --- | --- | --- | --- | --- |
| ebi-a-GCST90001991 | IBD | MR Egger | 40.87588 | 19 | 0.002507 | 0.001651205 | 0.016986265 | 0.923579 |
| ebi-a-GCST90001658 | IBD | MR Egger | 25.85365 | 29 | 0.633301 | 0.005729247 | 0.005492796 | 0.305544 |
