# Supplementary material for: Causal role of immune cells in inflammatory bowel disease: A Mendelian randomization study
Source: Medicine (Baltimore). 2024 Apr 5;103(14):e37537. doi: 10.1097/MD.0000000000037537 (PMC10994490; doi:10.1097/MD.0000000000037537)
Supplement: Supplementary file 3 [file medi-103-e37537-s005.docx]

**Supplementary Table 3** Exploring the causal impact of IBD on immune cell traits

| **traits** | **id** | **method** | **b** | **se** | **pval** | **lo_ci** | **up_ci** | **or** | **or_lci95** | **or_uci95** |
| --- | --- | --- | --- | --- | --- | --- | --- | --- | --- | --- |
| CD39+ CD4+ %T cell | ebi-a-GCST90001658 | Inverse variance weighted | 0.027269 | 0.03424 | 0.425802 | -0.03984 | 0.094379 | 1.027644 | 0.960941 | 1.098976 |
| HLA DR on CD14+ monocyte | ebi-a-GCST90001991 | Inverse variance weighted | -0.04511 | 0.056317 | 0.423091 | -0.1555 | 0.065268 | 0.955888 | 0.85599 | 1.067445 |
